# FHL3 promotes pancreatic cancer invasion and metastasis through preventing the ubiquitination degradation of EMT associated transcription factors

**DOI:** 10.18632/aging.102564

**Published:** 2020-01-13

**Authors:** Pengping Li, Guodong Cao, Yuefeng Zhang, Jingbo Shi, Kailun Cai, Lei Zhen, Xiaobo He, Yizhao Zhou, Yongzhou Li, Yi Zhu, Maoming Xiong, Yulian Wu

**Affiliations:** 1 The First People's Hospital of Xiaoshan Hangzhou, Zhejiang 311000, China; 2 Department of Surgery, Second Affiliated Hospital, Zhejiang University School of Medicine, Hangzhou, Zhejiang 310000, China; 3 Department of Gastrointestinal Surgery, Department of General Surgery, The First Affiliated Hospital of Anhui Medical University, Hefei, Anhui 230000, China; 4 Department of Hepatobiliary Surgery, Renmin Hospital of Wuhan University, Wuhan 430000, Hubei Province, China

**Keywords:** GSK3β, FHL3, EMT, metastasis, LIM-3 domain

## Abstract

Pancreatic ductal adenocarcinoma (PDAC) is intractable due to its strong invasiveness and metastatic ability. Epithelial-mesenchymal transition (EMT) is the pivotal driver of tumor invasion and metastasis. The four-and-a-half LIM domain (FHL) family is involved in regulating transforming growth factor (TGF)-β and Ras signaling, which might control the EMT process. In this study, we found that higher expression of four-and-a-half LIM domains 3 (FHL3) predicted poor prognosis in PDAC. The decreasing of FHL3 changed the EMT phenotype by blocking the TGFβ/Atk/GSK3β/ubiquitin pathways. Interestingly, the GSK3β inhibitor could abrogate the role of FHL3 in the regulation of snail1 and twist1 expression, which implied that GSK3β plays a pivotal role in the FHL3-mediated EMT process. Furthermore, we found that FHL3 can directly bind to GSK3β, which weakened the interaction between GSK3β and snail1/twist1. We also found that the LIM-3 domain of FHL3 was required for the binding of FHL3 to GSK3β. Collectively, our study implied that FHL3, as a binding partner of GSK3β, promoted tumor metastasis in PDAC through inhibiting the ubiquitin-degradation of snail1 and twist1.

## INTRODUCTION

Pancreatic ductal adenocarcinoma (PDAC) is an extremely malignant disease that is rarely diagnosed in an early stage, and the 5-year survival rate is lower than approximately 15% [1]. The surrounding tissue invasion characteristic of PDAC is an obstacle for clinical R0 resection, and chemotherapy or radiotherapy regimens cannot efficiently curtail the invasion lesions [2]. Therefore, a study of metastatic mechanisms is still needed to restrain PDAC invasion and metastasis.

Epithelial-mesenchymal transition (EMT) is a process by which epithelial cells lose apical-basal polarity and gain invasive ability [3]. The EMT process is a major initiator of tumor invasion and metastasis, and EMT could be activated by many EMT-associated transcription factors (EMT-TFs), such as snail1/2, twist1/2, and zeb1/2. Generally, down regulation of the expression of epithelial markers, such as E-cadherin (E-CAD), cytokeratins and occludins, and upregulation of the expression of mesenchymal markers, including N-cadherin, vimentin, matrix metalloproteases (MMPs) and α-SMA, are the characteristics of the process [3–6]. In fact, these EMT-TFs exactly correlate with lesion progression and chemoresistance in PDAC.

Four-and-a-half LIM domain (FHL) proteins, including FH1, FHL2, FHL3 and FHL5, are characterized by four evolutionarily conserved LIM domains and one conserved LIM superfamily domain [7]. As an actin-binding protein, FHL interacts with transcription factors and multiple cell signaling molecules, such as transforming growth factor (TGF) β/smad [8–11], Ras [12, 13], Wnt/β-catenin [14], and cell cycle process molecules [15, 16] to regulate cell proliferation, invasion and chemoradiotherapy resistance in tumors. Previous studies have shown that FHL acts as a tumor repressor in liver cancer, lung cancer, gastric cancer, and breast cancer [17–20]. However, FHL promotes paclitaxel resistance in liver cancer cells [21], EMT in breast cancer cells [22] and radioresistance in HeLa cells [15]. FHL3 was first found in skeletal muscle undergoing wound healing [23]. FHL3-mediated tumor growth has also been reported in glioma, breast cancer and liver cancer [9, 18, 24]. However, the relationship between FHL3 and tumor EMT remains unclear.

In this study, we investigated FHL3 expression in 49 paired PDAC samples. Then, we explored the effects of FHL3 on the EMT process and the underlying mechanisms of FHL3 in pancreatic cancer (PC) cell lines. Our aims were to clarify the role of FHL3 in oncogenesis in PDAC and to clarify the relationship between FHL3 and EMT.

## RESULTS

### FHL3 expression correlated with PDAC progression

**Figure 1:**
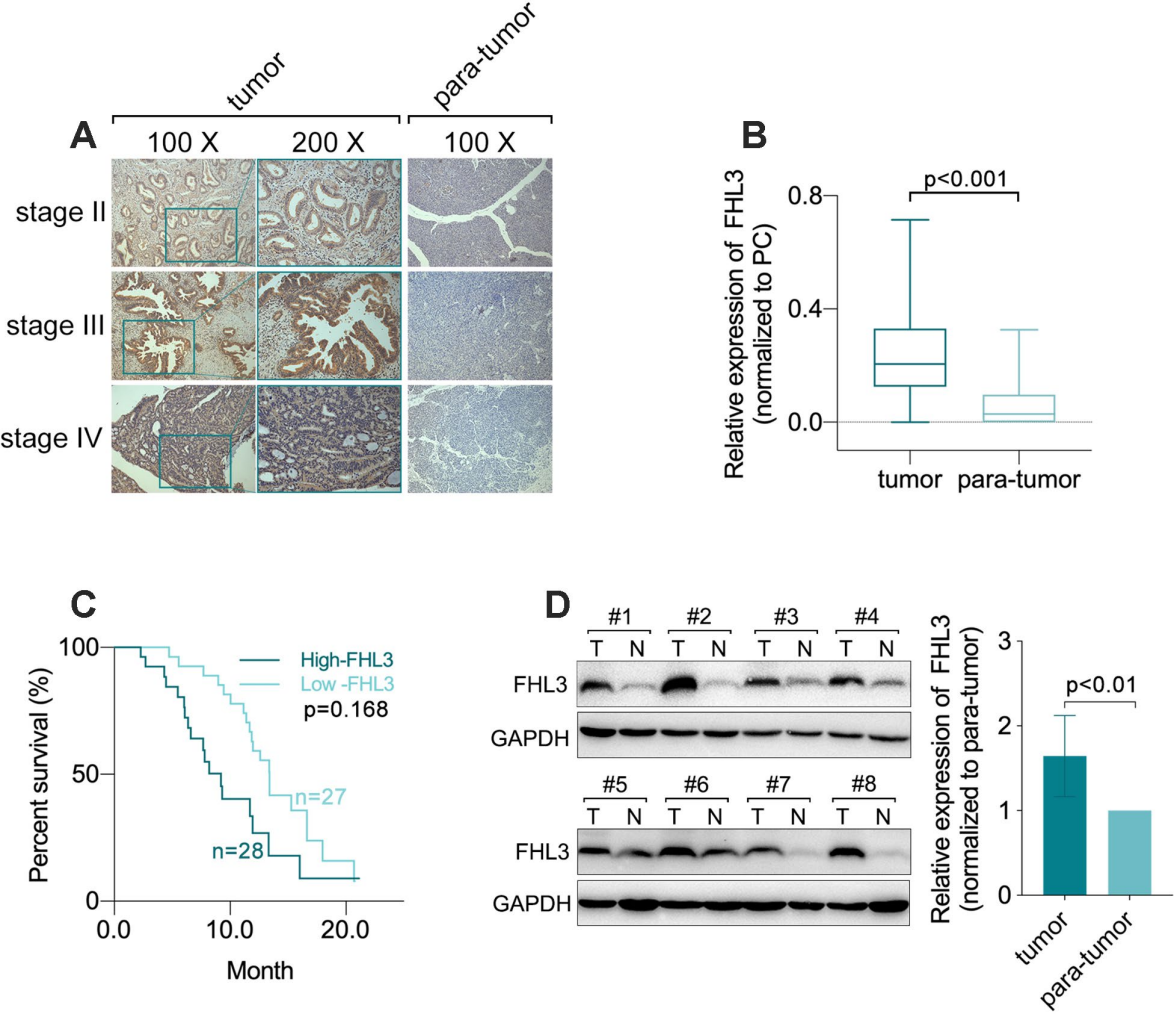
**FHL3 referred to PDAC progression.** (**A** and **B**) IHC staining and statistical analysis of FHL3 for sections from 55 matched different lesion stages tissues of PDAC and para-tumor normal area, which showed the higher expression level of FHL3 in PDAC, more than 3-fold, as compared with normal tissue, p<0.001. (**C**) Survival analysis via Kaplan-Meier analysis in 55 PDAC samples which showed higher expression level of FHL3 was accompanied with worse prognosis, p=0.0168. (**D**) WB assay of 8 matched fresh frozen PDAC samples, which showed higher expression level of FHL3 in PDAC tissues more than 1.5-fold, p<0.01.

In our study, we found that there was no significant difference in FHL3 expression in different groups stratified by age (p=0.304, Table 1), gender (p=0.912, Table 1), differentiation grade (p=0.342, Table 1) or M stage (p=0.826, Table 1). However, the quantification of immunohistochemistry (IHC) with Image-Pro^R^ Plus for 55 paraffin-embedded sections of PDAC showed that a higher expression level of FHL3 correlated with a higher clinical stage in PDAC (Figure 1A). And as Table.1 showed, higher expression level of FHL3 was accompanied with higher T stage (p=0.0165; 7 (26%) T1, 18 (67%) T2, and 2 (7%) T3 tissue sections in the low-FHL3 group; 2 (7%) T1, 16 (57%) T2 and 10 (36%) T3 tissue sections in the high-FHL3 group; Table 1) and N stage (p=0.0437; 22 (81%) N0 and 5 (19%) N1 tissue sections in the low-FHL3 group; 15 (54%) N0 and 13 (46%) N1 tissue sections in the high-FHL3 group; Table 1). In addition, FHL3 was overexpressed in PDAC tissue compared with its expression in adjacent non-tumor tissue in 49 paired paraffin-embedded sections of PDAC samples (p<0.001, Figure 1A and 1B), as same as the outcome of WB in eight matched fresh frozen PDAC samples (p<0.01, Figure 1D). Furthermore, the results of a Kaplan-Meier analysis of 55 PDAC samples grouped by the FHL3 expression measured by IHC indicated that higher expression of FHL3 implied worse prognosis in PDAC (p=0.0169, Figure 1C). In addition, multivariate analysis implied age (p<0.001), tumor site (p<0.025), T stage (p<0.015) and diabetes (p<0.020) made effects on prognosis (Table 2).

**Table 1:** Effect of FHL3 in progression of pancreatic cancer.

		**Expression level of FHL3**		***P*-Value**
		**Low**	**High**	
Age	>45	27	27	0.304
	<46	0	1	
Gender	Male	11	11	0.912
	Female	16	17	
Differentiation Grade	1	1	2	0.342
	2	2	2	
	3	16	10	
	4	8	14	
T Stage	1	7	2	0.016*
	2	18	16	
	3	2	10
N Stage	0	22	15	0.044*
	1	5	13	
M Stage	0	20	20	0.826
	1	7	8	
Overall survival	month	13.4	9.2	0.0168*

All 55 samples are pancreatic adenocarcinoma, and 26 of them are clinical stage II, the 29 of them are clinical stage III/IV (14 of stage III, 15 of stage IV).

*Statistically significant (P<0.05)

**Table 2:** Univariate and multivariate analysis of clinicopathological variables and FHL3 expression associated with overall survival

**Parameters**	**Univariate**			**Multivariate**	
	**HR (95% CI)**	***P*-value**		**HR (95% CI)**	***P*-value**
Age	0.039(0.004~0.433)	0.008*		0.384(0.171~.860)	<0.001*
Gender	0.879(0.452~1.708)	0.703		0.533(0.245~1.159)	0.112
Tumor site	0.855(0.570~1.284)	0.450		0.047(0.003~0.679)	0.025*
Differentiation grade	0.780(0.518~1.175)	0.235		1.054(0.652~1.705)	0.830
T Stage	0.986(0.601~1.616)	0.955		0.535(0.324~0.884)	0.015*
N Stage	1.421(0.700~2.884)	0.331		0.527(0.272~1.021)	0.058
M Stage	1.352(0.664~2.754)	0.406		0.848(0.358~2.008)	0.707
FHL3 expression	2.189(1.132~4.232)	0.020*		2.099(0.823~5.349)	0.120
Diabetes	0.760(0.413~0.760)	0.413		6.978(2.510~19.395)	0.020*

*Statistically significant (P<0.05)

### FHL3 expression was upregulated in pancreatic cancer cell lines and was associated with the metastasis ability

**Figure 2:**
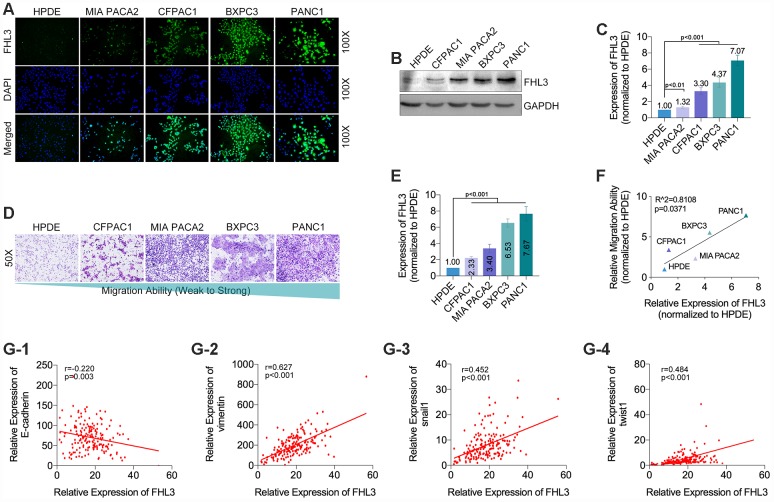
**The expression level of FHL3 referred to EMT makers and metastasis in pancreatic cancer cells.** (**A**) IF assay showed the expression level of FHL3 in four pancreatic cell lines and one normal pancreas cell line, and the expression rank was: PANC1>BXPC3>CFPAC1>MIA PACA2>HPDE. (**B** and **C**) WB assay showed the same results, and PANC1 and BXPC3 held the highest expression level of FHL3, which were more than 4-fold higher when compared to HPDE, p<0.001. (**D** and **E**) transwell assay for evaluating the migration ability in four pancreatic cell lines and one normal pancreas cell line, in which the migration rank was: PANC1>BXPC3>MIA PACA2>CFPAC1> HPDE. (**F**) linear relationship fitting analysis showed migration ability was correlate with the expression level of FHL3, r=0.8108, p=0.0371. TCGA data analysis showed FHL3 was negatively correlated to expression level of EMT maker E-cadherin (**G1**) (r=-0.220, p=0.003), and passively correlated to EMT maker vimentin (**G2**)(r=0.627, p<0.001) and EMT associated transcription factors snail1 (**G3**) (r=0.452, p<0.001, Fig.2G_3_) and twist1 (**G4**) (r=0.484, p<0.001).

Next, our study showed that, comparing with HPDE (normal pancreatic ductal epithelial cells), FHL3 expression was upregulated in four PC cell lines (PANC1, BXPC3, MIAPACA2 and CFPAC1) (Figure 2A–2C), and the highest upregulation of FHL3 was up to 7.07 folds (Figure 2C). As the same time, we found that higher expression levels of FHL3 in four PC cell lines and one normal pancreatic ductal epithelial cell line (HPDE<MIAPACA2< CFPAC1< BXPC3< PANC1) correlated with stronger migration ability (HPDE< CFPAC1<MIAPACA2<BXPC3<PANC1, Figure 2D–2F) through linear relationship fitting (r=0.8108, p=0.0371; Figure 2F). In addition, through analyzing the data in TCGA, our study found that expression of FHL3 was negatively correlated to expression level of EMT maker E-cadherin (r=-0.220, p=0.003, Figure 2G_1_), and passively correlated to EMT maker vimentin (r=0.627, p<0.001, Figure 2G_2_) and EMT associated transcription factors snail1 (r=0.452, p<0.001, Figure 2G_3_)and twist1 (r=0.484, p<0.001, Figure 2G_4_).

### FHL3 knockdown reversed the EMT phenotype and inhibited migration in pancreatic cell lines

**Figure 3:**
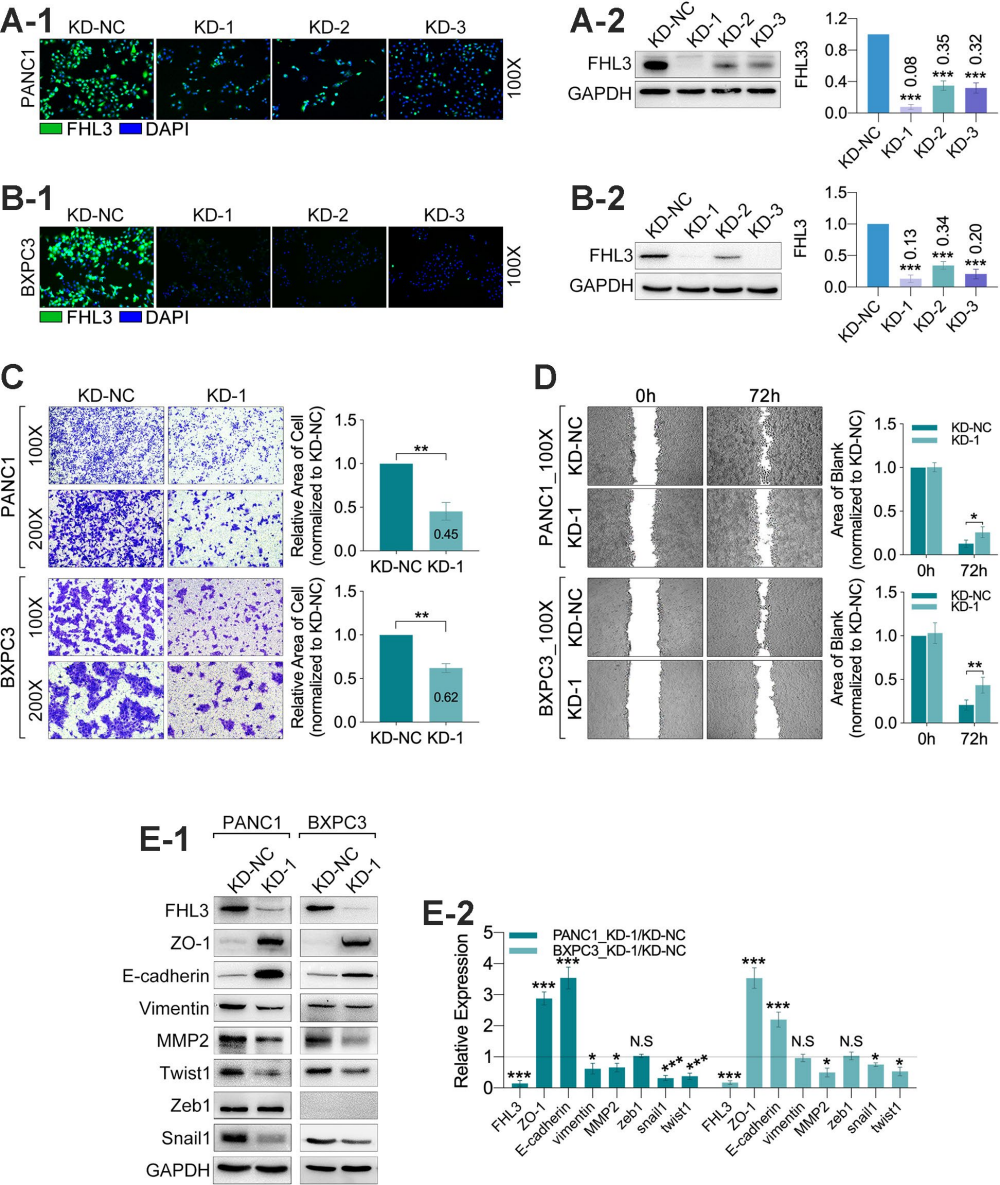
**FHL3 knockdown restrained migration and reversed EMT phenotype in pancreatic cancer cells.** (**A1** and **B1**) IF assay of FHL3 in PANC1_NC/KD_1-3_, BXPC3_NC/KD_1-3_, which showed KD1 sequence was the most efficient. (A2 and B2) WB assay of FHL3 in PANC1_NC/KD_1-3_ and BXPC3_NC/KD_1-3_, which showed the expression level of FHL3 was downregulated more than 80% in PANC1_KD1 and BXPC3_KD1 cells, p<0.001. (**C**) 48h- transwell assay showed more than 40% decreasing of migration ability of PANC1_KD1 and BXPC3_KD1 cells, as compared with PANC1_NC and BXPC3_NC cells, P<0.01. (**D**) 72h-wound healing assay showed KD1 cell lines held more blank area as compared with NC cell lines, p<0.05. (**E1**) WB assay showed FHL3 knockdown reversed the expression level of EMT associated proteins and transcriptional factors, except zeb1.

For finding out the internal connection between FHL3 and PDAC invasion, our study found the FHL3-knockdown (FHL3-KD) cell lines in PANC1 and BXPC3 (PANC1_KD1 and BXPC3_KD1). As Figure 3A_1_–2B_2_ showed, whatever the IF or WB, all of the three sequences for FHL3 knockdown were efficient, and the KD-1 cell lines (PANC1 and BXPC3) were the most efficient in FHL3 knockdown, for PANC1 was 92% (p<0.001, Figure 3A_1-2_)and for BXPC3 was 87% (p<0.001, Figure 3B_1_-_2_). So, in the following study, we chose the KD-1 cell lines as our objects. As Figure 3C showed, FHL3 knockdown inhibited the migration of pancreatic cells, in which the migration inhibition rate was 55% in PANC1_KD1 (p<0.01, Figure 3C) and 38% in BXPC3_KD1 (p<0.01, Figure 3C). In another one experiment, named wound healing assay, our study found the lower expression of FHL3 was accompanied with the larger blank area, which mean the weaker ability of metastasis, in PANC1 (P<0.05, Figure 3D) and BXPC3 (p<0.01, Figure 3D) cell lines. We confirmed that the efficient knockdown of FHL3 expression in PANC1_KD1 and BXPC3_KD1 cells resulted in significant upregulation of ZO-1 (PANC1_KD1: about 3 fold, p<0.001; BXPC3_KD1: about 3.5 fold, p<0.001; Figure 3E_1-2_) and E-cadherin (PANC1_KD1: about 3.5 fold, p<0.001; BXPC3_KD1: about 2.5 fold, p<0.001; Figure 3E_1-2_), and obvious downregulation of MMP2 (PANC1_KD1: about 40%, p<0.05; BXPC3_KD1: about 50% fold, p<0.05; Figure 3E_1-2_), snail1 (PANC1_KD1: about 70%, p<0.001; BXPC3_KD1: about 20% fold, p<0.05; Figure 3E_1-2_), twist1 (PANC1_KD1: about 60%, p<0.001; BXPC3_KD1: about 40% fold, p<0.05; Figure 3E_1-2_). However, the knockdown of FHL3 just made downregulation of vimentin in PANC1_KD1 (about 40%, p<0.05, Figure 3E_1-2_), but not in BXPC3_KD1. Meantime, the expression of zeb1 was unchanged after FHL3 knockdown.

### FHL3 regulated EMT process through TGFβ1/Akt/GSK3β/ubiquitin process but not through TGFβ1/smad_2/3_/smad4 pathway

**Figure 4:**
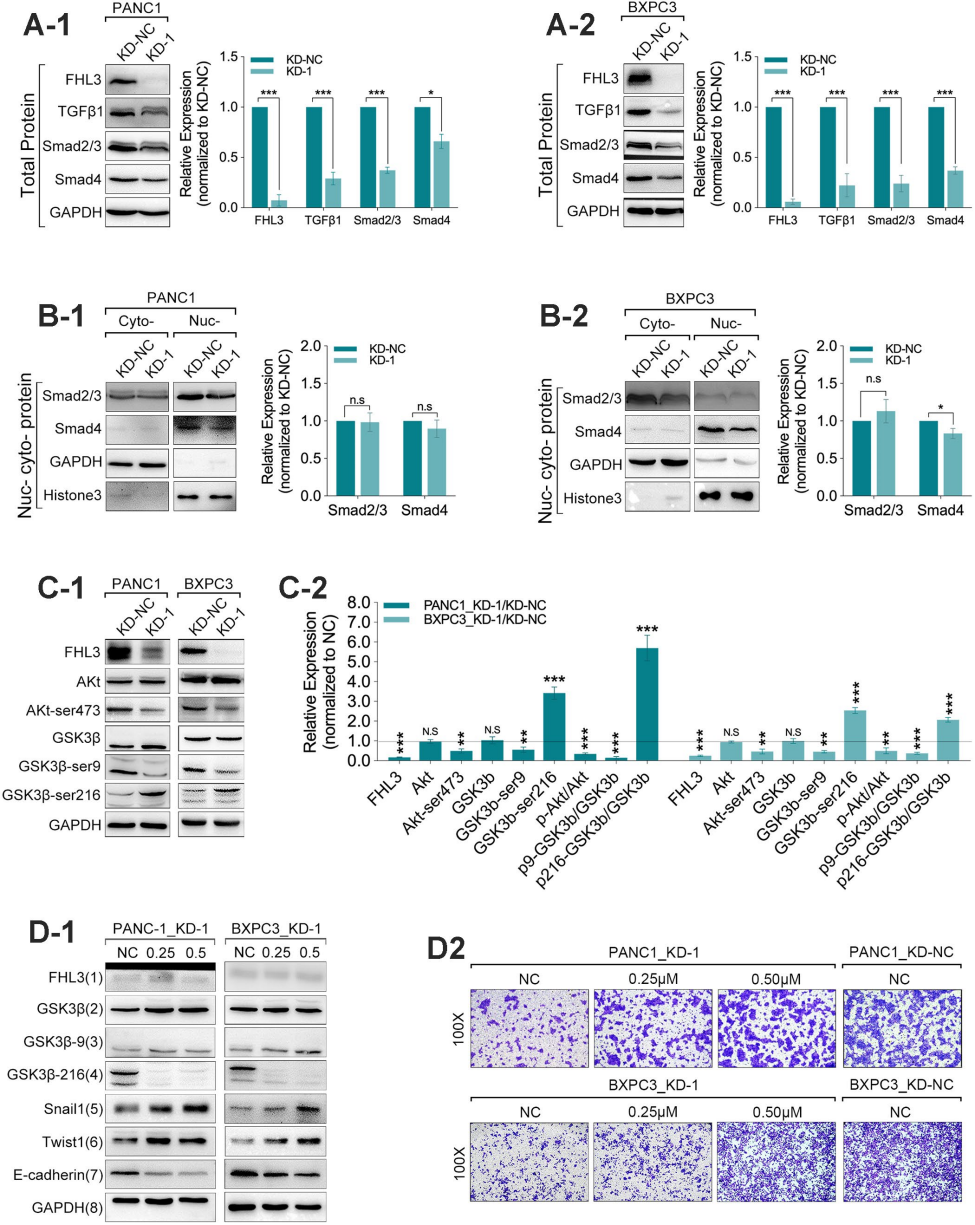
**FHL3 regulated EMT mainly by TGFβ1/Akt/GSK3β/ubiquitin pathway.** (**A1** and **A2**) WB assay showed FHL3 knockdown made downregulation of TGFβ1, smad_2/3_, and smad_4_ in PANC1_KD1 and BXPC3_KD1 in total protein level. (**B1** and **B2**) WB assay of nucleus-cytoplasm protein showed that FHL3 knockdown hardly changed the expression level of smad_2/3_ and smad_4_ in nucleus in PANC1_KD1 and BXPC3_KD1 cells. (**C1** and **C2**) In PANC1_KD1 and BXPC3_KD1 cells, FHL3 knockdown exactly downregulated the absolute and relative expression of phosphorylated AKT (ser473-AKT) more than 50%, p<0.01; and also downregulated the absolute and relative expression of phosphorylated GSK3β (ser9-GSK3β) more than 50%, p<0.01; and upregulated the absolute and relative expression of phosphorylated GSK3β (try216-GSK3β) more than 2-fold, p<0.001. (**D1**) 0.25μM and 0.50μM GSK3β inhibitor almost eliminated the effect, promoting the TGFβ1/AKT/GSK3β/ubiquitin process, caused by FHL3 knockdown. As treated with GSK3β inhibitor, GSK3β (try216-GSK3β) and E-cadherin were downregulated, snail1 and twist1 were upregulated. (**D2**) 0.25μM and 0.50μM GSK3β inhibitor reversed the migration ability of PANC1_KD1 and BXPC3_KD1 cells.

Next, our study found that the FHL3 knockdown made significant downregulation of TGFβ1 (>60% in PANC1_KD1 and BXPC3_KD1, p<0.001, Figure 4A_1-2_), smad2/3 (>50% in PANC1_KD1 and BXPC3_KD1, p<0.001, Figure 4A_1-2_) and smad4 (about 50% in PANC1_KD1 and BXPC3_KD1, p<0.001, Figure 4A_1-2_). However, our study found there was almost no influence in the expression of smad_2/3_ in nucleus/cytoplasm rate in PANC1 and BXPC3 cell lines, and just not more than 20% decreasing of smad_4_, in nucleus/cytoplasm rate, as FHL3 knockdown (Figure 4B_1-2_). As previous studies implied, snail1 was regulated by GSK3β-mediated ubiquitin degradation. Therefore, we explored the following research. In the following study, we found FHL3 knockdown changed the TGFβ1/Akt/GSK3β/ubiquitin pathway. As Figure 4C_1-2_ showed, FHL3 knockdown not only decreased the absolute level of phosphorylated Akt (ser473-Akt) more than 50%, but also relative level of phosphorylated Akt (ser473-Akt/Akt) more than 50% in PANC1_KD1 and BXPC3_KD1. Meantime, FHL3 knockdown was accompanied with more than 50% downregulation of phosphorylated GSK3β (ser9-GSK3β) and more than 2-fold upregulation of phosphorylated GSK3β (try216-GSK3β), whatever in absolute expression level or relative expression (p-GSK3β/GSK3β) (Figure 4C_1-2_). This study implied that FHL3 knockdown decreased the TGFβ1 level, weakened the activity of Akt, result of which further enhanced the activity of GSK3β. In order to verify our study, we use GSK3β inhibitor, 1-Azakenpaullone, for next experiments. On the one hand, as Figure 4D_1_ showed, the activity of GSK3β was decreased with the increasing dose of inhibitor both in PANC1_KD1 and BXPC3_KD1. And, the level of snail1 and twist1 were also increased after treatment of GSK3β inhibitor (Figure 4D_1_). Finally, EMT maker E-cadherin was significantly downregulated (Figure 4D_1_). On the other hand, GSK3β inhibitor absolutely reversed the pancreatic cells migration ability which was blocked by FHL3 knockdown both in PANC1_KD1 and BXPC3_KD1 (Figure 4D_2_).

### FHL3 competitively binded to GSK3β by LIM-3 domain to inhibit ubiquitin process for maintaining the level of EMT associated transcriptional factors

**Figure 5:**
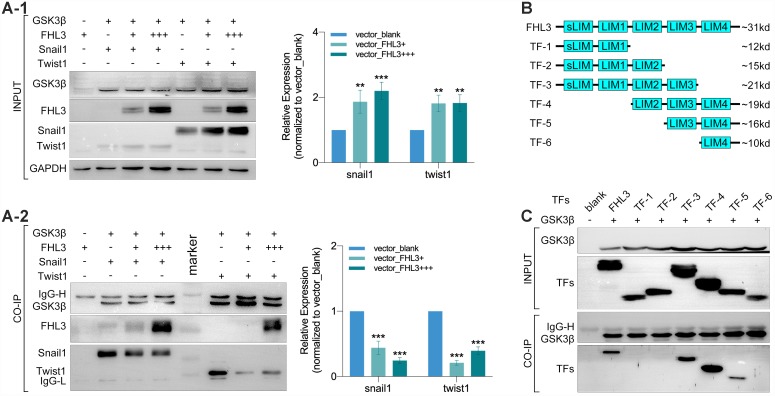
**LIM-3 was the pivotal domain for FHL3 competitively binding to GSK3β.** (**A1** and **A2**) Transfection with GSK3β, FHL3 (+:5ug, +++:15ug), snail1 and twist1 in HEK293T for 48h followed by CO-IP assay, which showed the higher FHL3 expression was accompanied with the higher expression of snail1 and twist1, p<0.01; meantime, the higher FHL3 expression made less snail1 and twist1 which binding with GSK3β, p<0.001. (**B**) Truncated forms form FHL3. (**C**) Transfection with GSK3β and truncated forms for 48h in HEK293T cells, and the CO-IP assay showed only truncated forms which containing LIM-3 domain could bind with GSK3β.

In order to thoroughly determine the roles of FHL3 in the ubiquitin mediated degradation process of EMT associated TFs, we explored the physical interactions between FHL3 and proteins in the ubiquitin process. Coimmunoprecipitation (CO-IP) and mass analysis showed that FHL3 could interact with GSK3β (data not provided) and E3 ligase (RNF146) (data not provided). Based on these results, we performed experiments in HEK293T cells. As our study showed, the expression level of snail1 and twist1 were upregulated about 2-fold with an increased transfection dose of the FHL3-HA plasmid (0μg, 5μg and 15μg, Figure 5A_1_). Furthermore, as the transfection dose of the FHL3-HA plasmid increased, more FHL3 bound to GSK3β with less snail1/twist1 bound to GSK3β (decreased more than 50%, Figure 5A_2_); few FHL3 molecules bound to the negative control (Figure 4A_2_). These results implied that FHL3 was directly involved in the ubiquitin mediated degradation of snail1 and twist1 through competitively binding to GSK3β to weaken the interaction between GSK3β and snail1/twist1. Furthermore, we explored the GSK3β binding ability of the pivotal domain in FHL3. We designed six truncated forms, as shown in Figure 4B, and transfected plasmids into HEK293T cells. Our study showed that only TF-3, TF-4 and TF-5, all of which contained the LIM-3 domain, could bind to GSK3β. In addition, due to LIM-3 domain deletion, TF-1, TF-2 and TF-6 could not bind to GSK3β (Figure 5C). These results implied the LIM-3 domain was required for FHL3 binding to GSK3β.

### FHL3 knockdown curbed pancreatic cancer cells growth and metastasis in vivo

**Figure 6:**
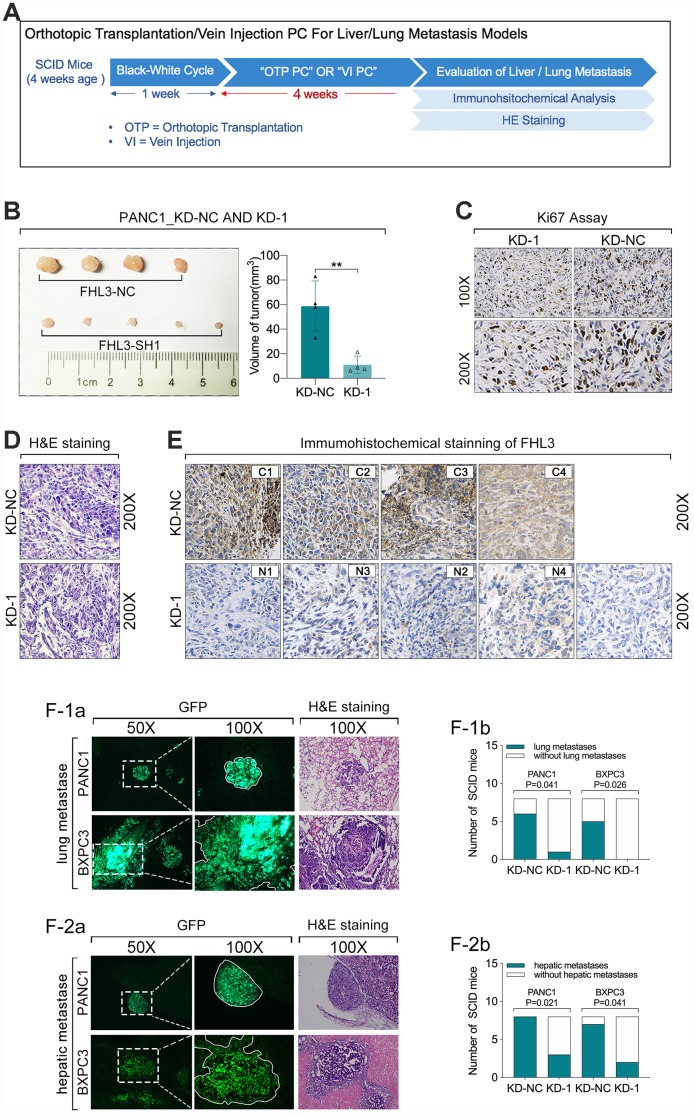
**FHL3 knockdown inhibited pancreatic tumor growth and metastasis in vivo.** (**A**) experiments process in vivo. (**B**) tumor volume of PANC1_KD1 and PANC1_NC, 4 weeks after orthotopic transplantation; and the tumor volume of PANC1_KD1 group was about 1/3 of PANC1_NC group, p<0.01. (**C**) FHL3 knockdown made the weaker Ki67 staining in tumor sections of PANC1_KD1 group. (**D**) H&E staining of tumor section. (**E**) IHC staining of FHL3 of tumor section. (**F1a**, **F1b**) lung metastasis from circular pancreatic tumor cells, and FHL3 knockdown made the lower occurrent rate of lung metastasis in PANC1_KD1 and BXPC3_KD1 groups, p<0.05. (**F2a**, **F2b**) hepatic metastasis from orthotopic transplantation tumor, and FHL3 knockdown made the lower occurrent rate of hepatic metastasis in PANC1_KD1 and BXPC3_KD1 groups, p<0.05.

Then, we validated the role of FHL3 in growth and migration of pancreatic cancer cells in vivo. As Figure 6A showed, 4 weeks after the orthotopic transplantation or tail intravenous injection of pancreatic cancer cells, the liver and lungs were harvested for tumor detection. During our study in vivo, there was a SCID mice died of unknown cause. In our result, we found orthotopic transplantation tumor of PANC1_KD1 cell was smaller more than 50% as compared with PANC1_NC cell (p<0.01, Figure 6B). And HIC staining of Ki67 for those tumor slices showed that PANC1_KD1 cell tumor held stronger staining signal than PANC1_NC cell tumor (Figure 6C and Supplementary Figure 1). In addition, we also verified the expression level of FHL3 by HIC, which implied PANC1_KD1 cells exactly significantly lose the FHL3 (Figure 6E and Supplementary Figure 1). Next, in the tumor metastasis model experiments, as Figure 6F_1-a, b_ showed, green area were metastatic tumor from circular pancreatic cancer cells, which were from tail vein injection, and lung metastasis occurred in 1 of the 8 SCID mouse in PANC1_KD1 cells (12.5%), which occurred in 6 of 8 SCID mouse in PANC1_NC cells (75%), that mean FHL3 knockdown decreased the lung metastasis from circular tumor cells more than 50%. And we got the same result in BXPC3_KD1 cells (Figure 6F_1-a, b_). During the experiments of hepatic metastasis model, our study found occurrent rate of hepatic metastasis was 100% (8/8) in PANC1_NC cells group and 87.5% in BXPC3_NC cells group with which was only 37.5% in PANC1_KD1 cells group and 25% in BXPC3_KD1 cells group (Figure 6F_2-a, b_). Those data showed that FHL3 maintained the invasion and metastasis ability in pancreatic cancer cell lines.

## DISCUSSION

Pancreatic ductal adenocarcinoma (PDAC) is a malignant tumor with strong invasive ability and few early diagnosis techniques, which make for tough obstacles to PDAC treatment. FHL proteins, which mediate protein-protein interactions, play paradoxical roles in tumor growth, invasion and chemoradiotherapy resistance. However, in our study, we showed that 1. FHL3 was a biomarker of progression in PDAC; 2. FHL3 plays a role in tumor growth; and 3. FHL3 promotes metastasis by upregulating the expression of EMT associated transcription factors in pancreatic cancer cell lines.

EMT is a pivotal driver of tumor metastasis, which is characterized by the upregulation of the expression of MMPs, vimentin, N-cadherin and α-SMA, accompanied by the downregulation of the expression of E-cadherin, occludins and cytokeratin [3–6]. The mechanism that regulates EMT is complex, containing the Wnt/β-catenin pathway, Akt/GSK3β/ubiquitin pathway, TGFβ pathway, and Ras pathway [25–27]. EMT-TFs such as snail1/2, zeb1/2, and twist1/2 are the terminal targets of all of the above pathways.

LIM domain-containing proteins, including the FHL family and lim only protein (LMO) family, both of which consist of only evolutionarily conserved LIM domains, play paradoxical roles in tumors. As previous studies have shown, FHL can restrain the expression of cyclinA/B/D/E, upregulate p21 and p27 expression, or inhibit the effect of CDC25 by directly binding to CDC25, and all of these effects initiate G1/2 phase arrest, which endows chemoradiotherapy resistance [12, 15, 16].

In addition, FHLs can enhance the transcriptional activation of TGF-β and smad_2/3/4_, and it can also directly enhance the phosphorylation of smad_2/3_ with the assistance of CK1δ, all of which increases the nuclear translocation of the smad_2/3/4_ complex [9]. The upregulated TGFβ pathway promotes EMT-TF expression dependent on smad_2/3/4_ at the transcriptional level [25]. In our study, our data showed the downregulation of TGFβ, smad_2/3_ and smad_4_ expression in the total cell lysates (Figure 4A_1-2_), but there were few changes in nuclear translocation of smad_2/3/4_ (Figure 4B_1-2_), both effects of which were triggered by FHL3 knockdown. We found weaker metastatic ability in pancreatic cancer cells (PANC1_KD1 and BXPC3_KD1 cells), both in vitro and in vivo experiments, after FHL3 knockdown (Figure 3C, 3D; Figure 6F_1-2_). These results suggest that FHL3 regulated EMT process and tumor metastasis were not through TGFβ pathway.

However, we found that the mRNA levels of snail1 and twist1 were upregulated (data were not showed here), rather than downregulated, after FHL3 knockdown (data was not in here). Therefore, there was another pathway by which FHL3 regulated the expression of snail1 and twist1. As previous studies showed, FHLs can strengthen Akt expression and activity at the transcriptional level in TGFα-dependent and TGFα-independent ways [28]. Furthermore, FHLs is involved in Ras signaling [12, 13], which is linked to complex cell biology processes, including the PI3K/Akt/mTOR, Wnt/β-catenin, and TGFβ/smad pathways [29]. Furthermore, GSK3β, a downstream target of these pathways, has been shown to be a pivotal player in snail1 and twist1 degradation [26, 27, 30]. Therefore, based on the above findings, our study explored the roles of FHL3 in the ubiquitin-mediated degradation of snail1 and twist1. As our study showed, FHL3 knockdown slightly downregulated Akt expression and significantly weakened Akt activity by strongly abrogating the phosphorylation of ser473 (Figure 4C_1-2_). Accordingly, the enhanced activity of GSKβ was shown by the upregulation of the phosphorylation of try216 and the downregulation of ser9, accompanied by the upregulation of snail1 and twist1 expression (Figure 3E_1-2_), which was also validated in the context of FHL3 overexpression (Figure 5A_1_). Furthermore, we also found that the ability of FHL3 to regulate snail1 and twist1 was almost completely eliminated by a GSK3β inhibitor (Figure 4D_1-2_). Furthermore, we found that FHL3 could interact with GSK3β and that FHL3 could compete with snail1 and twist1 to bind to GSK3β in concentration-dependent ways (Figure 5A_2_). Then, we found that LIM-3 was the pivotal domain which was required for the combination of FHL3 and GSK3β, and LIM-3 domain might mediate the physical interaction between snail1/twist1, GSK3β and FHL3 (Figure 5C). Therefore, we believe that FHL3 enhanced the stability of snail1 and twist1 through the TGFβ/Akt /GSK3β/ubiquitin pathway.

The upregulation of TGFβ/smad signaling can inhibit tumor growth in early phase of tumor, but promote tumor progression in middle-later phase of tumor. And, previous studies have shown the tumor-growth-inhibition effects of FHL1 and FHL2 was mediated by TGFβ/smad signaling [9]. In addition, FHL2 can also promote tumor growth by upregulating Ras signaling [12]. In our study, we found that decreased TGFβ/smad signaling failed to promote the tumor growth, instead of restraining tumor growth, induced by FHL3 knockdown.

**Figure 7:**
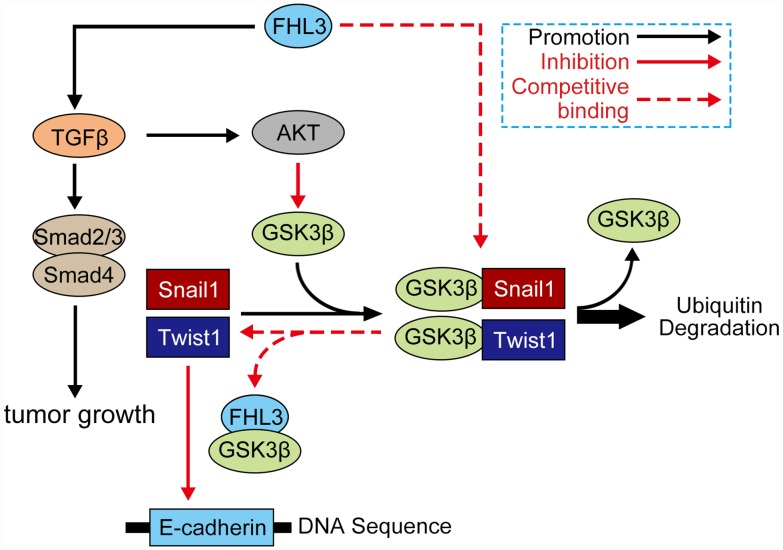
**Mechanism of FHL3 in regulation of EMT process.** Protein which involved in signaling: AKT (AKT, ser437-AKT), GSK3β (GSK3β, ser9-GSK3β, try216-GSK3β), FHL3, snail1, twist1, TGFβ1, smad (smad_2/3_, smad_4_), and E-cadherin.

Generally, our study showed the following highlights: 1) FHL3 lead to PDAC progression; 2) FHL3 maintained tumor growth in pancreatic cancer; 3) FHL3 elevated EMT-TFs to promote EMT process through the TGFβ/Akt/GSK3β/ubiquitin pathways but not TGFβ/smad_2/3/4_ pathway (Figure 7). In addition, we believe that FHL3 may be a risk factor for PDAC and that the LIM-3 domain may be used to restrain PDAC metastasis, which will supply a new treatment strategy for PDAC.

## MATERIALS AND METHODS

Purpose for this study is to explore the roles of FHL3 in proliferation and metastasis about pancreatic cancer. All experiments were repeated, respectively, at least three times for gaining reliable data.

### Reagents

SB525334, 1-Azakenpaullone, Bortezomib were purchased from Shelleck (USA), and dissolved in DMSO. Recombinant Human Transforming Growth Factor β1 (TGFβ1) was purchased from Proteintech (China), and saved in sterile deionized water within 50% glycerin. Gemcitabine was purchased from Ely Lilly (Bad Homburg, Germany) and dissolved in sterile 0.9% sodiumchloride. Bovine serum albumin (BSA) was purchased from Sigma-Aldrich.

### Pancreatic cancer samples preparation

This study was approved by The First Affiliated Hospital of Anhui Medical University Review Board and the ethics committees of Anhui Medical University. 49 matched paraffin-embedded tissue sections, 6 tumor paraffin-embedded tissue sections and 8 paired fresh frozen tissue were collected from tissue bank from January 2011 to January 2018. All patients with pancreatic ductal adenocarcinoma were confirmed by at least two pathologists.

### Cell culture

Pancreatic cancer cell lines (PANC1, MIAPACA2, CFPAC1, and BXPC3) and normal pancreas ductal epithelial cell (HPDE) were gained from the cell bank of Chinese academy of science in October 2017 with STR matching analysis. PANC1 and MIAPACA2 were cultured in DMEM (Gibco, USA), CFPAC1 was cultured in IMDM (Gibco, USA), BXPC3 and HPDE was cultured in RPIM-1640 (Gibco, USA). All types of culture media were supplemented with 10% fetal calf serum and 100 units/mL penicillin and streptomycin, but 20% fetal calf serum for CFPAC1.

### Cell proliferation and cytotoxicity assays

The cell proliferation was quantified by standard curve (0.1, 0.2, 0.4, 0.8, 1.0, 1.5, 2.0, 3.0×10^4^ cells were detected optical density (OD) via cell counting kit-8 (Japan) after 24h transplanted into 96-wells plates, and then fit linear standard curve between log [cell quantity] and OD), cell cytotoxicity assays was performed via MTT assay, and the detail protocol described in our previous study (PMID29331423 and PMID29800682).

### Quantitative real-time PCR (qRT-PCR)

Trizol RNA solation system (Invitrogen, USA) was used for total RNA extraction. The cDNA templates were synthesized through PrimeScript RT Reagent Kit (TaKaRa, China), and qRT-PCR was performed with a 7500 Fast™ System (Applied Biosystems, USA) using the Sensi Mix SYBR Kit (Bio-Rad, USA). The mRNA level was calculated via using (=2^-ΔΔCt^), and normalized to GAPDH. All of the sequences of primer were designed by Primer 5 soft, see in Supplementary Table 1.

### Western blot analysis

### Total protein extraction

Cells were harvested by cytology brush, and lysed with RIPA lysis buffer (Sigma, USA) supplemented with phosphorylase and protease inhibitor mixture (Thermo, USA), quantified by the BCA assay.

### Cytoplasmic and nucleus protein extraction

Cells were harvested by Tyrisin (Invitrogen), then cytoplasmic and nucleus protein was extracted by Cytoplasmic and Nucleus Protein Extraction Kit (Thermal Scientific, USA) according to its protocol, quantified by the BCA assay.

The standard detail experimental process of western blot as same as our previous study (PMID29331423 and PMID29800682). Western blot band was quantified through the Image-J software (NIH, USA). Antibodies against GAPDH, FHL3, E-cadherin, MMP2 and GSK3β were purchased from Proteintech (1:1000, Hangzhou, China), antibodies against smad2/3, smad4, twist1, Ubiquitin, Histone 3, ser473-phospho-Akt, try216/279-phospho-GSK3β, ser9-phospho-GSK3β and ser423/425-phospho-smad2/3 were purchased from Huabio (1:1000, Hangzhou, China), antibodies against ZO-1, Akt, vimentin, snail1 and zeb1 were gained from Abcam (1:1000, China).

### Small interfering RNA (siRNA) and recombination plasmid (RP)

### Small interfering RNA (siRNA) experiments

5 × 10^5^ pancreatic cancer cells were transplanted into 6 wells plates for 24h, and then cells were transfected with three different sequences FHL3 siRNA (GenePharma, Shanghai, China) for 48h, 72h and 96h with Lipofectamine 3000 reagent (Invitrogen, USA) and Opti-MEM (Life Technologies, USA), according to the manufacturer's instructions for gaining the best transfection efficiency. Three siRNA sequences for FHL3 were listed in Supplementary Table 2.

### Recombination plasmid experiments

Primers of FHL3, GSK3β, Snail1, Twist1 and RNF146, inserted into plasmid pcDNA 3.1(-) (Addgene), were designed with Primer 5 soft, see in Supplementary Table 3. Briefly, cDNA templates were synthesized through PrimeScript RT Reagent Kit (TaKaRa, China); CDS of genes were amplified with PrimeSTAR® GXL DNA Polymerase (TaKaRa, China); thirdly, products were purified through SanPrep Column DNA Gel Extraction Kit (Sangon Biotech, China); fourthly, the purified products and plasmid were treated with restriction endonuclease (Xho1, EcoR5 and Xba1 were purchased from NEB, USA) respectively; fifthly, recombination of plasmids were performed through homologous recombination with Hieff Clone^TM^ Plus One Step Cloning Kit (Yeasen Biotech, China). All primers were listed in Supplementary Table 3. 5 × 10^5^ cells were transplanted into 6 wells plates for 24h, and then cells were transfected with RP for 48h, 72h and 96h with Hieff Trans^TM^ Liposomal Transfection Reagent (Yeasen Biotech, China) for the best transfection efficiency, according to the manufacturer's instructions.

### Immunofluorescence analysis

Briefly, 2.5 × 10^4^ pancreatic cancer cells were seeded in 24-well plates for 24h, and then fixed by 4% paraformaldehyde, permeabilized by 0.5% Triton X-100, and blocked with 5% bovine serum albumin (BSA, Sigma) for 1 h at 37 °C. Samples were incubated with primary antibody (FHL3, 1:200, Proteintech) overnight at 4°C. Subsequently, it washed by PBS, incubated with secondary antibodies for 1h in room temperature before being washed again. Finally, nuclei were stained with 15 μl DAPI (Sigma, USA) before detected by fluorescence microscope (Carl Zeiss, Germany).

### Immunohistochemistry staining and scoring standard

Experiments procedure of paraffin embedding, tissue section, hematoxylineosin (HE) staining and immunohistochemistry for FHL3 expression level were performed as previously described (PMID: 23200678 and 20571492). What more, the work concentration of antibody against FHL3 (Proteintech, China) was 1:150, and 1:200 for Ki67 proliferation index (Abcam). The protein expression level was assessed by Mean of Integrated Option Density (IOD) with Image-Pro^R^ Plus. Briefly, all of the Immunohistochemical sections were photographed for three yields in the same standard, and then select Area of Interesting (AOI) and detect IOD to gain Mean of IOD (IOD/AOI, MI), normalized to positive control (vascular smooth muscle cells). Finally, FHL3 expression level was divided into high and low group according to Mean of MI.

### Immunoprecipitation

1×10^7^ Cells were harvested by cytology brush, and lysed with RIPA lysis buffer (Yeasen Biotech, 20118ES60) for protein supernatant, followed by adding immune magnetic beads (Anti-Myc, Anti-HA and Anti-Flag, Bimake) for continuous slight mixing in 4°C for 24h. And then gain immune magnetic beads with Magnetic frame (Bimake), followed by TBS washing. Finally, products were boiled before dissolved in 5x SDS (Yeasen) for 5-10 minutes for western blot assay.

### Migration ability assay

Migration ability assays contain transwell and wound healing assay. For transwell, 5 × 10^4^ cells, with special treatments or not, were transplanted into transwell plates (24-well, 8.0μm, Corning Incorporated, Corning, NY, USA) with 10% gradient of fetal calf serum for 48h. And the detection procedure was same as our previous study (PMID29331423). Quantification of passed cell area was performed by Image-Pro^R^ Plus. For wound healing assay, cells were seeded at least 90% fusion in 6-well plates, and scratched by 200ul pipette tip, then washed with PBS to remove shed cells for extra 96h culture (PMID29331423). Scratch area was quantified with Image-Pro^R^ Plus.

### Experimental protocols in vivo

### Tumor growth experiments in vivo

Female athymic nude mice (4 weeks), gained from the SLAC (Shanghai, China), were randomly divided into four groups. 1 × 10^6^ cells (PANC1_NC/KD1), in 100ul PBS, were injected into the tail of pancreas. All mouse was sacrificed and the orthotopic pancreatic tumors were harvested for detecting tumor volume (MaA×MiA^2^ / 2; MaA=Major axis, MiA=Minor axis), and followed by being processed into frozen sections for HE staining, immunofluorescence staining and Ki67 staining.

### Tumor metastasis experiments in vivo

Female SCID mice (4 weeks), gained from the SLAC (Shanghai, China), were randomly divided into eight groups. 1 × 10^6^ cells (PANC1_NC/KD1, BXPC3_NC/ KD1), in 100ul PBS, were injected into pancreas tail vein for orthotopic-liver-metastasis tumor model, then all mouse were sacrificed and livers were harvested for HE staining and immunofluorescence staining after 4 weeks. For lung metastasis experiments, 1 × 10^6^ cells (PANC1_NC/KD1, BXPC3_NC/KD1), in 100ul PBS, were injected into tail vein, then all mouse was sacrificed and lungs were harvested for HE staining and immunofluorescence staining after 4 weeks.

### Statistics

All experimental data were presented as the means ± SD. Statistical Package for the Social Sciences version 21.0 (SPSS Inc., USA) was used for statistical analyses. ANOVA, paired t-test, Chi-square (*x*^2^) test and nonparametric test (Mann Whitney U) for statistical analysis of different situations. Statistical significance was considered when p < 0.05 (*p < 0.05; **p < 0.01; ***p < 0.001). All histograms and curves were constructed with GraphPad Prism 6 software (GraphPad Software, La Jolla, CA, USA).

## Supplementary Material

Supplementary Figure 1

Supplementary Tables
