# Supplementary material for: FHL3 promotes pancreatic cancer invasion and metastasis through preventing the ubiquitination degradation of EMT associated transcription factors
Source: Aging (Albany NY). 2020 Jan 13;12(1):53–69. doi: 10.18632/aging.102564 (PMC6977653; doi:10.18632/aging.102564)
Supplement: Supplementary Figure 1 [file aging-12-102564-s001..pdf]

SUPPLEMENTARY FIGURE

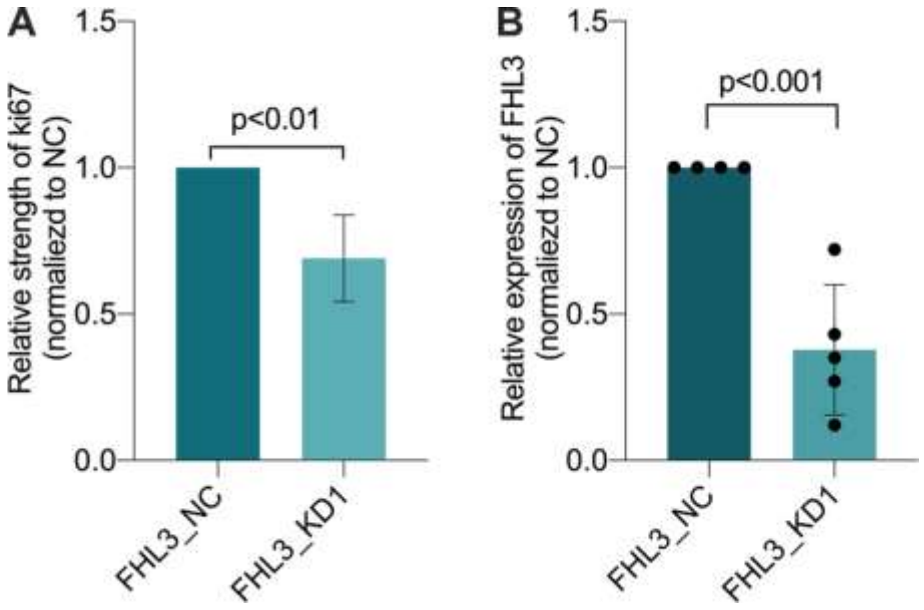

Supplementary Figure 1. FHL3 knockdown inhibited tumor growth in vivo. (**A**) Statistic of Ki67 staining strength of tumor sections from PANC1_KD1 and PANC1_NC, p<0.01. (**B**) Statistic of FHL3 IHC of tumor sections from PANC1_KD1 and PANC1_NC, p<0.001.
