# Supplementary material for: FHL3 promotes pancreatic cancer invasion and metastasis through preventing the ubiquitination degradation of EMT associated transcription factors
Source: Aging (Albany NY). 2020 Jan 13;12(1):53–69. doi: 10.18632/aging.102564 (PMC6977653; doi:10.18632/aging.102564)
Supplement: Supplementary Tables [file aging-12-102564-s002..pdf]

SUPPLEMENTARY TABLES

Supplementary Table 1. Nucleotide sequences of primers for qRT-PCR.

Gene		Primers (from 5' to 3')
FHL3	Forward	GGACGCAAGTACATCCAGACAGAC
	Reverse	GGCACTCAGAGGTGTTGG
ZEB1	Forward	GAGGAGGAGGAGGAGGAAGAAGTG
	Reverse	CACTTGCTCACTCTCGCCTAC
SNAI1	Forward	GGCTCCTTCGTCCTTCTCCTCTAC
	Reverse	CCAGGCTGAGGTATTCCTTGTTGC
GAPDH	Forward	ATGGGGAAGGTGAAGGTCGGAGTC
	Reverse	TTACTCCTTGGAGGCCATGTGGGC
TWIST1	Forward	GACTTCCTCTACCAGGTCCTCCAG
	Reverse	TCCAGACCGAGAAGGCGTAGC

Supplementary Table 2. FHL3-siRNA sequence in this study.

Sequence Name		Sequence
FHL3 -405	Sense	GCCGAUGAACCCUUCACCUTT
	Anti-sense	AGGUGAAGGGUUCAUCGGCTT
FHL3 -535	Sense	GCCAGACAUGGCAUGAGCATT
	Anti-sense	UGCUCAUGCCAUGUCUGGCTT
FHL3 -707	Sense	CCGUGAUCAGCCGUGGCAUTT
	Anti-sense	AUGCCACGGCUGAUCACGGTT

Supplementary Table 3. Primers of inserted sequence in recombination plasmid.

Gene		Primer (from 5' to 3')
FHL3-HA	Sense	AGCGTTTAAACGGGCCCTCTAGCCACCATGAGCGAGTCATTTGACTGTGCAA
	Anti-sense	TGGTGGAATTCTGCAGATATCTCAAGCGTAGTCTGGGACGTCGTATGGGTAGGGCCCTGCCTGGCTA CAGCCCT
Snail1-Flag	Sense	AACGGGCCCTCTAGACTCGAGGCCACCATGCCGCGCTCTTTCCTCGTCA
	Anti-sense	TGGTGGAATTCTGCAGATATCTCACTTATCGTCGTCATCCTTGTAATCGCGGGGACATCCTGAGCAGC CG
Twist1-Flag	Sense	AACGGGCCCTCTAGACTCGAGGCCACCATGATGCAGGACGTGTCCAG
	Anti-sense	TGGTGGAATTCTGCAGATATCTCACTTATCGTCGTCATCCTTGTAATCGTGGGACGCGGACATGGACC
GSK3β-Myc	Sense	AACGGGCCCTCTAGACTCGAGGCCACCATGTCAGGGCGGCCCAGAACCA
	Anti-sense	TGGTGGAATTCTGCAGATATCTCACAGATCCTCTTCAGAGATGAGTTTCTGCTCGGTGGAGTTGGAA GCTGATGCAG
RNF146-Myc	Sense	AACGGGCCCTCTAGACTCGAGGCCACCATGGCTGGCTGTGGTGAAATTGAT
	Anti-sense	TGGTGGAATTCTGCAGATATCTCACAGATCCTCTTCAGAGATGAGTTTCTGCTCAACTTCAGTTACTG TGCACTGTCC
